# Systemic effects of hypophosphatasia characterization of two novel variants in the *ALPL* gene

**DOI:** 10.3389/fendo.2023.1320516

**Published:** 2024-01-03

**Authors:** Luis Martínez-Heredia, Manuel Muñoz-Torres, Raquel Sanabria-de la Torre, Ángela Jiménez-Ortas, Francisco Andújar-Vera, Trinidad González-Cejudo, Victoria Contreras-Bolívar, Sheila González-Salvatierra, José María Gómez-Vida, Cristina García-Fontana, Beatriz García-Fontana

**Affiliations:** ^1^Instituto de Investigación Biosanitaria de Granada, Granada, Spain; ^2^Endocrinology and Nutrition Unit, University Hospital Clínico San Cecilio, Granada, Spain; ^3^Department of Medicine, University of Granada, Granada, Spain; ^4^Biomedical Research Network in Fragility and Healthy Aging (CIBERFES), Instituto de Salud Carlos III, Madrid, Spain; ^5^Department of Biochemistry, Molecular Biology III and Immunology, University of Granada, Granada, Spain; ^6^Department of Biochemistry and Molecular Biology II, University of Granada, Granada, Spain; ^7^Department of Computer Science and Artificial Intelligence, University of Granada, Granada, Spain; ^8^Andalusian Research Institute in Data Science and Computational Intelligence (DaSCI Institute), Granada, Spain; ^9^Bioinformatic Service, Instituto de Investigación Biosanitaria de Granada, Granada, Spain; ^10^Clinical Analysis Unit, University Hospital Clínico San Cecilio, Granada, Spain; ^11^Pediatric Unit, University Hospital Clínico San Cecilio, Granada, Spain; ^12^Department of Cell Biology, University of Granada, Granada, Spain

**Keywords:** hypophosphatasia, tissue non-specific alkaline phosphatase, autoimmune diseases, gastrointestinal disorders, metabolic disease

## Abstract

**Introduction:**

Hypophosphatasia (HPP) is an inborn metabolic error caused by mutations in the ALPL gene encoding tissue non-specific alkaline phosphatase (TNSALP) and leading to decreased alkaline phosphatase (ALP) activity. Although the main characteristic of this disease is bone involvement, it presents a great genetic and clinical variability, which makes it a systemic disease.

**Methods:**

Patients were recruited based on biochemical assessments. Diagnosis was made by measuring serum ALP and pyridoxal 5-phosphate levels and finally by Sanger sequencing of the ALPL gene from peripheral blood mononuclear cells. Characterization of the new variants was performed by transfection of the variants into HEK293T cells, where ALP activity and cellular localization were measured by flow cytometry. The dominant negative effect was analyzed by co-transfection of each variant with the wild-type gene, measuring ALP activity and analyzing cellular localization by flow cytometry.

**Results:**

Two previously undescribed variants were found in the ALPL gene: leucine 6 to serine missense mutation (c.17T>C, L6S) affecting the signal peptide and threonine 167 deletion (c.498_500delCAC, T167del) affecting the vicinity of the active site. These mutations lead mainly to non-pathognomonic symptoms of HPP. Structural prediction and modeling tools indicated the affected residues as critical residues with important roles in protein structure and function. In vitro results demonstrated low TNSALP activity and a dominant negative effect in both mutations. The results of the characterization of these variants suggest that the pleiotropic role of TNSALP could be involved in the systemic effects observed in these patients highlighting digestive and autoimmune disorders associated with TNSALP dysfunction.

**Conclusions:**

The two new mutations have been classified as pathogenic. At the clinical level, this study suggests that both mutations not only lead to pathognomonic symptoms of the disease, but may also play a role at the systemic level.

## 1 Introduction

**Figure 1:**
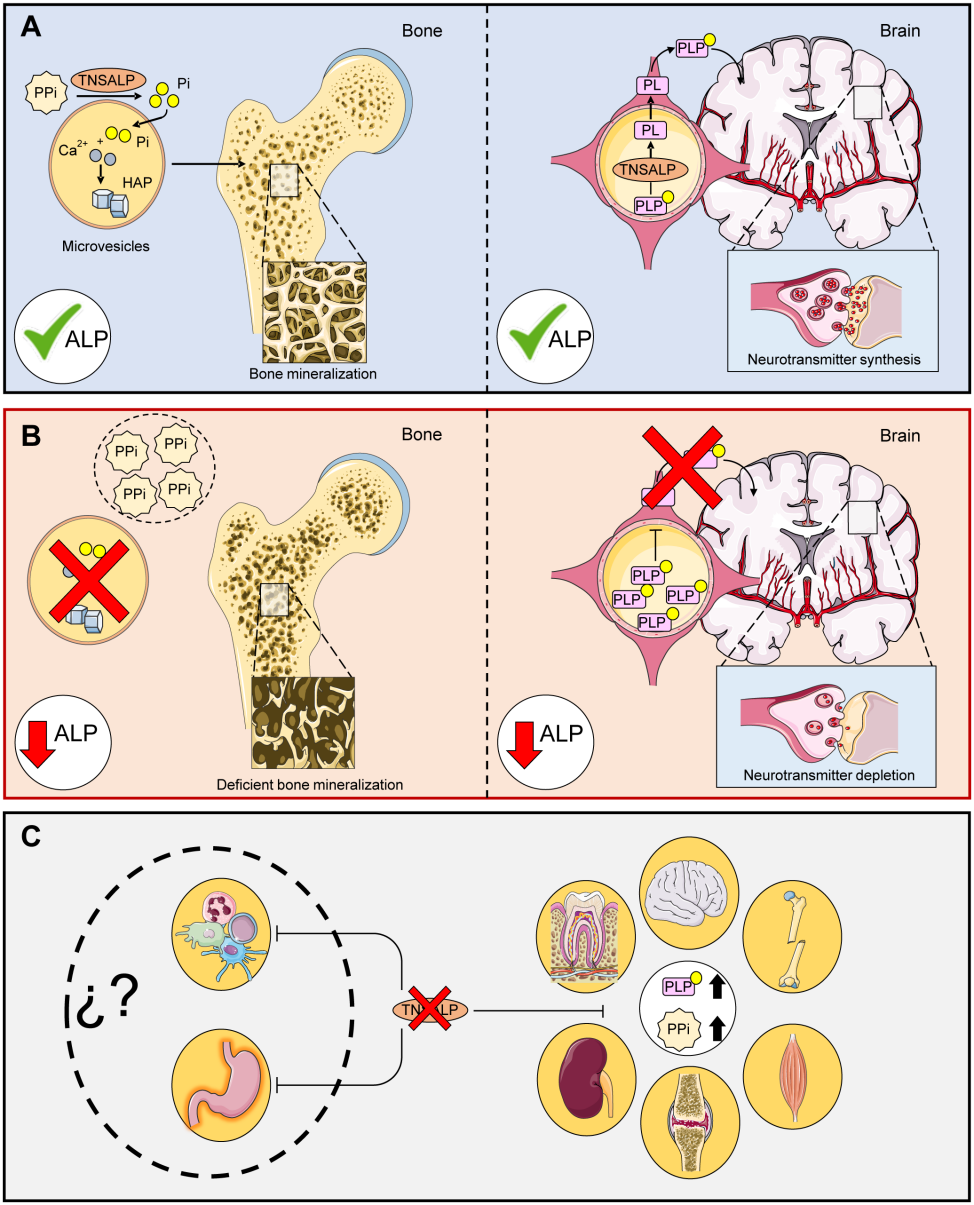
Main functions of TNSALP in the body. **(A)** shows the correct function of TNASLP: PPi is hydrolyzed to Pi for subsequent conversion to hidroxyhapatite crystals (HAP) in microvesicles via the action of TNSALP. PLP is hydrolyzed to PL by TNSALP to cross the blood-brain barrier, where it is subsequently reconstituted into PLP and leads to the formation of neurotransmitters. **(B)** shows the molecular effects related to defective TNSALP: Loss of function in TNSALP produces an accumulation of its substrates. PPi inhibits bone mineralization while PLP cannot cross the blood-brain barrier, decreasing the production of neurotransmitters. **(C)** shows clinical manifestations related to defective TNSALP: The accumulation of PPi alters calcium/phosphate homeostasis, causing bone, tooth, renal and joint damage, while increased levels of PLP lead to a decrease in B6 supply at the neurological level, which can lead to seizures, muscle and respiratory problems. However, the autoimmune and digestive implications have not been explored to date.

Hypophosphatasia (HPP) is a rare genetic and, in some cases, a lethal disease characterized mainly by bone and tooth mineralization defects (1). This disease is caused by one or more loss-of-function mutations in the *ALPL* gene encoding tissue non-specific alkaline phosphatase protein (TNSALP) (2). The prevalence of this disease is difficult to estimate due to the lack of knowledge of this disorder leading to a high rate of underdiagnosis. In 2011, Mornet et al. reported a mild HPP prevalence of 1/6,370 for Europe (3). However, our research group, in 2019, indicated that the prevalence of mild HPP in Spain may be higher than previously thought (1/3,100) (4). In 2021, Mornet et al. reevaluated the prevalence of mild HPP in Europe (1/2,430), which aligned with the prevalence we observed in Spain (5). Nevertheless, our most recent study, published in 2023, has revealed a new estimate for mild HPP prevalence in Spain, nearly double the earlier figure (1/1,692 vs. 1/3,100) (6). During the last 20 years, the perception of this disease has evolved significantly from a rare, recessive, bone disease to a systemic disease with higher incidence than previously reported and dominant inheritance in the mild forms (7).

TNSALP is a homodimer ectoenzyme (8) belonging to the alkaline phosphatase (ALP) family (EC 3.1.3.1). There are four other tissue-specific phosphatases in this family: intestinal (IALP), placental (PALP) and germ cell (GCALP) alkaline phosphatases, which are encoded by the *ALPI*, *ALPP* and *ALPP2* genes respectively. These enzymes hydrolyze monoester bonds from their substrates to yield inorganic phosphate (Pi). TNSALP acts on inorganic pyrophosphate (PPi) to yield Pi, one of the substrates to produce hydroxyapatite crystals. Pyridoxal-5-phosphate (PLP), also known as vitamin B6, is another major substrate of TNSALP and a precursor of some neurotransmitters. This molecule is hydrolyzed to pyridoxal (PL) to cross the blood-brain barrier where it will be reconverted to PLP (2, 9) (**Figure 1A**). Other TNSALP substrates described include lipopolysaccharide (LPS) (10), adenosine triphosphate (ATP) (11) and phosphorylated osteopontin (12).

TNSALP is involved in numerous pleiotropic processes (13, 14) and is expressed in most tissues and organs according to the Human Protein Atlas (Web References), particularly in bone, kidney and liver. The most frequent clinical manifestation of HPP is high rates of bone fragility and fractures as well as neurological disorders. This is due to the accumulation of their substrates that inhibit bone mineralization and lead to a decrease in neurotransmitter precursors (**Figure 1B**). In addition, some forms of rickets and osteomalacia have been linked to deficiencies in this enzyme (15, 16). However, these clinical features do not apply to all HPP patients due to the vast variety of mutations as well as the different types of inheritance. The clinical features of this disease range from completely asymptomatic patients to complete absence of bone mineralization and fetal death (17). The disease conditions can affect different organs and systems including skeletal (17, 18), muscular (19), dental (20), neurological (21), respiratory (17), renal (22) and articular (23) due to accumulation of their substrates (**Figure 1C**).

There are more than 500 described variants in the *ALPL* gene according to the Leiden Open Variation Database (LOVD) website, 628 nonsynonymous variants in The Genome Aggregation Database (gnomAD) website and 438 nucleotide variants and 14 structural variants in ALPL mutation database (Web References) which strongly contributes to the wide phenotypic heterogeneity of this disease. In addition, many of these variants have a dominant negative effect (DNE), which further exacerbates this phenotypic variability. DNE is defined as the decrease below 50% of TNSALP activity when the wild-type (WT) allele and the mutated allele are co-expressed at the same levels (24, 25). Due to these factors, is very difficult to establish a relationship between genotype and phenotype in HPP patients. In this context, del Angel et al. (2020), using structural model predictions in silico algorithms, observed associations between residual enzymatic between residual enzyme activity and variant origin, variant type, affected protein domain, and HPP subtype (26). However, although there appears to be a relationship between low *in vitro* TNSALP levels and disease severity, there are other variables that influence the development of clinical manifestations in affected patients.

In this study, two previously undescribed mutations are presented in two patients recently diagnosed with childhood-onset HPP, with no familial relationship and with completely different clinical features. The aim is to characterize each of the new mutations at the genetic, structural and functional levels to establish a relationship with the clinical features. Clinical characteristics and biochemical parameters have been useful in establishing a correct diagnosis of the rest of the relatives. In this context, it is worth emphasizing the importance of establishing a geno-phenotypic relationship for each newly identified mutation to provide more information and better patient management.

## 2 Materials and methods

### 2.1 Patients

Two fifteen-year-old male patients were evaluated in the Endocrinology Unit of the University Hospital Clínico San Cecilio of Granada following the algorithm developed by García-Fontana et al. (4). Neither of the two patients took vitamin B6 supplements. Patients with secondary causes of hypophosphatasemia such as malnutrition, magnesium and zinc deficiencies, haemochromatosis or certain therapies, were excluded (2, 27). Two venous blood samples were taken from each patient at the Clinical Analysis Unit of the University Hospital Clínico San Cecilio; one was for ALP and PLP determinations, and the other one was used for *ALPL* gene sequencing. Written informed consent was obtained from their legal guardians and an individualized and personal interview was conducted on potentially related HPP symptoms. This study was approved by the ethics committee of Granada following the principles of the World Medical Association Declaration of Helsinki (Project ID: 0777-M1-20. Research Ethics Committee of Granada Center [(CEI-Granada) on 8 May 2019].

### 2.2 Clinical analysis

ALP activity was measured bichromatically at 410/480 nm by conversion of p-nitrophenyl phosphate to p-nitrophenol in the presence of magnesium, zinc and 2-amino-2-methyl-1-propanol as phosphate acceptor at pH 10.4 from blood samples on AU5800 analyzers (Beckman Coulter) according to the method recommended by the International Federation of Clinical Chemistry. ALP determinations were performed in the Clinical Analysis Laboratory of University Hospital Clínico San Cecilio. The reference values for fifteen-year-old males were 75-312 IU/L following the values indicated in the CALIPER study adjusted by age and sex (28).

Plasma PLP levels were measured by high-pressure liquid chromatography (HPLC) at the Clinical Unit of the University Hospital Niño Jesús (Madrid). Chromatographic determination was determined using an isocratic HPLC system. For PLP detection, an emission laser at 320 nm and a fluorescence detector was used. Reference values (3.6-18 ng/mL) were established by University Hospital Niño Jesús and for fifteen-year-old males.

The DNA used for sequencing was collected from peripheral blood lymphocytes and the polymerase chain reaction (PCR) of the *ALPL* gene was performed following the method described by Riancho et al. (29). The PCR product underwent Sanger sequencing and the canonical sequence (Gene ID: 249, RefSeq: NM_000478.6 (ALPL Transcript variant 1), UniProt: P05186-1) was used as a reference. The alternative *ALPL* transcripts and their corresponding protein sequences has been included in **Supplementary Tables S1, S2**. Finally, a copy variant number study was performed by multiple ligation probe amplification (MLPA) (MRCHolland) and the results were analyzed using the SeqPilot program (JSI Medical System). The Biomedical Diagnostic Center of the Clinic Hospital of Barcelona provided the sequencing results.

After confirmation of the presence of mutations in the *ALPL* gene in the two patients, available relatives were recruited to perform ALP blood measurements, interviews about their clinical history, and *ALPL* gene sequencing. According to de IFCC, the reference values for adult males were 43-115 IU/L while for females were 33-98 IU/L.

### 2.3 Sequence prediction and three-dimensional modeling of TNSALP

MutPred, PROVEAN and Mutation Taster algorithms were used to predict the consequences of protein mutations. Combined annotation-dependent depletion (CADD) was used to rank mutations according to impact and was compared to the mutation significance cut-off (MSC) obtained for CADD scores (30). Both mutations have already been introduced into the VarSome database with references NM_000478:c.17T>C for the L6S variant and NM_001369805.2:c.498_500del for the T167del variant.

To determine the degree of conservation of the amino acids affected by the new variants in the TNSALP protein of Homo sapiens (P05186-1), a multiple sequence analysis (MSA) was performed. Twenty species encompassing different types of vertebrates including different classes such as reptiles, amphibians, birds, fish and mammals belonging to different orders were chosen for the analysis. Among the different orders of mammals, rodents, artiodactyls, perissodactyls, carnivores and primates of the genera macaca, pongo, pan and gorilla were chosen (**Supplementary Table S3**). Multiple sequence alignment was performed using the ClustalW tool of the Unipro UGENE V.45.0 software (31).

For 3D modelling, the complete atomic model was predicted using AlphaFold2_advanced (32). The models with the highest scores in the local distance difference test (pLDDT) were chosen. Finally, the visualization and preparation of the figures were performed using Chimera X software (33).

### 2.4 Cell culture

Human embryonic kidney cells 293T (HEK293T) were used. Briefly, cells were cultured at 37°C and 5% CO_2_ with Dulbecco’s Modified Eagle Medium (DMEM) High Glucose (pH 7.2) (Biowest) supplemented with 10% fetal bovine serum (Capricorn scientific), 5% Ham’s F12 Nutrient Medium (Biowest) and 1% of 100X Antibiotic-Antimycotic (Biowest).

### 2.5 Plasmid design

The vectors used were constructed by modifying the pcDNA3.1 plasmid. The *ALPL* gene with the study variants L6S (pcDNA3.1:ALPL c.17T>C) and T167del (pcDNA3.1:ALPL c.498_500delCAC) was inserted in this plasmid. The pcDNA3.1 plasmid with the WT *ALPL* gene insertion (pcDNA3.1: ALPL) was used as a positive control to functionally characterize the identified variants. The empty vector (EV) without insertion (pcDNA3.1) was used as a negative control to monitor the basal expression of the *ALPL* gene at the cellular level. The different variants of the *ALPL* gene were inserted between the *HindIII* and *BamHI* restriction sequences belonging to the multi-cloning site. All vectors were supplied by GenScript.

### 2.6 Cell transfection

Before HEK293T cells transfection, 150000 cells were grown per well in 24-well plates. After 24 hours, transient transfection was performed by adding to each well 50 µL of serum-free DMEM containing 1.5 µL of LipoD293 DNA *in vitro* transfection reagent (SignaGen Laboratories) and 500 ng of the corresponding plasmid. Co-transfections were carried out by mixing 250 ng of each variant with 250 ng of WT obtaining 500 ng of total reaction. The cells were incubated for 18 hours with the mixture and after that time, 2 mL of DMEM supplemented with serum was added. Finally, the cells were incubated for 24 hours and harvested for the different assays. The term homozygous will refer to cells transfected with a single plasmid while the term heterozygous will refer to cells co-transfected unless otherwise indicated.

### 2.7 *ALPL* gene expression

RT-qPCR was performed to determine the exogenous levels of *ALPL* gene expression by each construction. Firstly, RNA was collected from each transfected culture using the RNeasy® Mini Kit (Qiagen) and treated with DNase (Qiagen). For cDNA synthesis, 600 ng of template RNA and the iScript cDNA synthesis kit (BioRad) were used following the manufacturer’s protocol. For quantitative PCR, PowerUP SYBR Green Master Mix (Thermo Fisher Scientific) was used in the CFX96 real-time thermal cycler (BioRad). Gene expression was normalized using the ribosomal protein L13 (RPL13) used as a constitutive gene. The set of primers used for the determination of *ALPL* expression is listed in **Supplementary Table S4**. Finally, the results were analyzed by using the ΔΔCt method.

### 2.8 Flow cytometry and antibody staining

Cell viability was determined by using FITC Annexin V Apoptosis Detection Kit I (BD Biosciences) following the manufacturer’s protocol. Cells that were negative for both propidium iodide and Annexin V expression were considered viable cells, while cells that were positive for Annexin V, individually or together with propidium iodide, were considered apoptotic cells.

Antigenic density was performed following the protocol developed by Lopez-Perez et al. (34). Briefly, the cells were washed with PBS and incubated with 2 µL of BV421 Mouse Anti-Human Alkaline Phosphatase (BD Biosciences) for 20 minutes. Then, the cells were fixed with 4% formaldehyde for 20 minutes. Finally, the cells were washed twice with PBS and resuspended in 100 µL. To standardize the expression of TNSALP variants on cell membranes, 5 µL of CountBright™ Absolute Count Beads (Invitrogen) were resuspended in 100 µL of PBS. All results were obtained with the BD FACSAria III Cell Sorter flow cytometer (BD Biosciences) on a logarithmic scale. Antigenic density was calculated as the ratio of the median intensities of the TNSALP-positive cells versus the median intensity obtained by the CountBrigh Absolute Count Beads.

### 2.9 Alkaline phosphatase activity

TNSALP activity was measured at a wavelength of 450 nm by spectrophotometry (Dynex Technologies) using the Alkaline Phosphatase Detection Kit (Abnova) from cell extracts according to the manufacturer’s recommended protocol.

### 2.10 Statistical analysis

Each experiment was performed in triplicate. Saphiro-Wilk test was used to test the normal distribution of data. To evaluate the differences between groups, the one-way ANOVA test was used followed by Tukey HSD. P-values below 0.05 were considered significant. All tests were conducted with GraphPad Prism 9.5.1.

## 3 Results

### 3.1 Determination of ALP and PLP levels and clinical manifestations

**Table 1** shows the results of the biochemical analyses of each patient. The blood ALP activity in Patient 1 (P1) and 2 (P2) had persistently low ALP activity (73 IU/L and 45 IU/L respectively) while the PLP concentration were 6.5 and 2.5 times higher (118 µg/L and 45.5 µg/L respectively) than the normal values. Regarding clinical manifestations, P1 showed symptoms related to digestive, neurological, endocrine and muscular systems, gastroesophageal reflux disease (GERD) and dyspepsia, *pes valgus* deformity, vitamin D deficiency and high blood pressure (HBP) while P2 presented clinical manifestations related to autoimmune diseases such as Crohn’s disease and inverse psoriasis in addition to vitamin D deficiency. P2 was treated with calciferol and immunosuppressants. Interestingly, none of the patients presented pathognomonic symptoms of HPP. After two years of follow-up, both patients maintained persistently low levels of ALP activity (P1: 42 IU/L; P2 38 IU/L).

**Table 1:** Anthropometric and clinical parameters of HPP patients.

	P1	P2
*ALPL* mutation	c.17T>C (L6S)	c.498_500delCAC (T167del)
ALP Activity (75-312 IU/L)	73/42	45/38
PLP (3.6-18 µg/L)	118	45.5
Age (years)	15	15
BMI (Kg/m^2^)	25.40	24.30
Serum 25-vit D (30-50 ng/mL)	12.20	19.80
Protein c-reactive (0-5 mg/L)	1.20	2.60
Interleucin-6 (1.5-7 pg/L)	3.30	1.50
Parathyroid Hormone (12-110 pg/mL)	83.80	49.90
Symptoms	myalgia, pes valgus deformity, GERD, vitamin D deficiency, HBP and cephalea.	Crohn’s disease, vitamin D deficiency, Inverse psoriasis, Asthenia

HBP, high blood pressure; GERD, gastroesophageal reflux disease.

### 3.2 Sequence analysis

**Figure 2:**
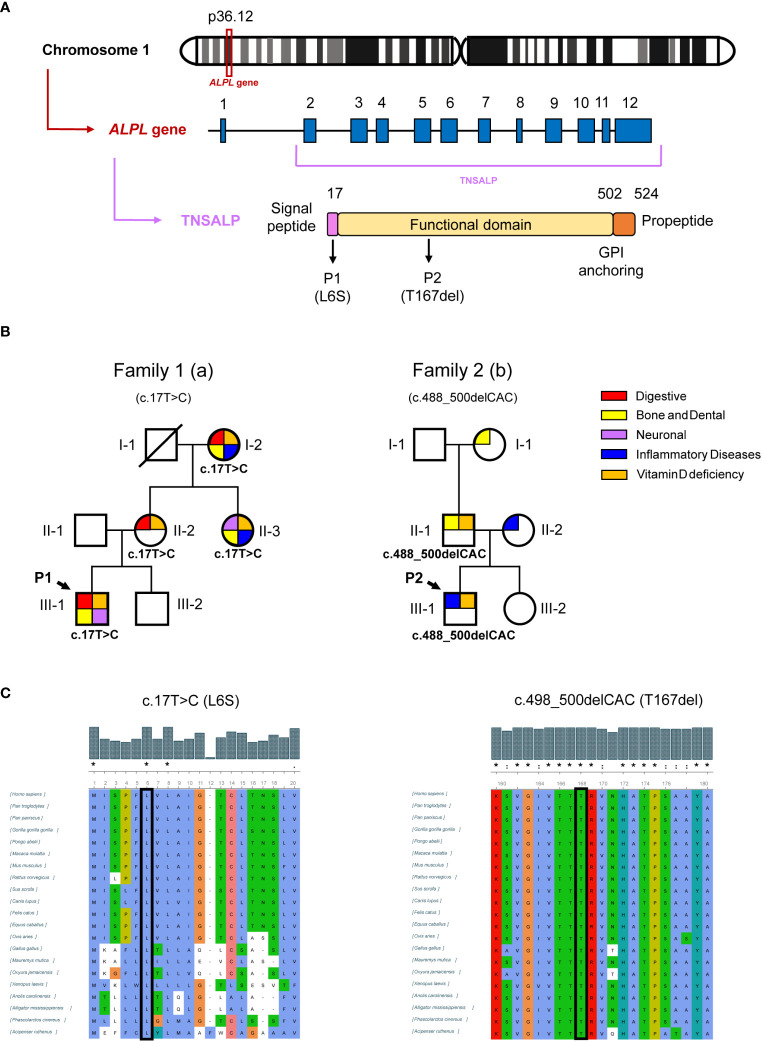
Genetic results of patients and relatives. **(A)** shows schematic representation of the location of the *ALPL* gene on chromosome 1, the *ALPL* gene with twelve exons and the TNSALP precursor protein with its N-terminal signal peptide, the functional domain, the C-terminal glycosylphosphatidylinositol (GPI) anchor binding site and the propeptide sequence. **(B)** shows genogram of the two families affected by HPP. The pathologies that affect each individual are summarized by categories. **(C)** shows the representation of the conservation of the human TNSALP mutated residues after MSA by ClustalW. The symbols (*,:.) represent identical, conserved and semi-conserved substitutions respectively while the absence of a symbol represents a lack of amino acid characteristic conservation. The whole alignment is shown in **Supplementary Material**.

P1 presented a missense mutation in heterozygosis in the second exon of the *ALPL* gene (c.17T>C) changing a leucine for serine in position 6 of the protein sequence (L6S). P2 was found to be heterozygous for a three-nucleotide in-frame deletion (c.498_500del) that resulted in the loss of the threonine residue at position 167 (T167del) in the TNSALP sequence. **Figure 2A** shows a schematic context of the newly identified variants at genomic, transcriptional and protein levels. Both mutations have not been previously described in the scientific literature as they have not been found in any database such as genomeAD LOVD or Clinical relevant Variation (ClinVar) (Web References).

The results of the pathogenicity predictions are shown in **Table 2**. P1 showed a disparity of results regarding the prognosis of the severity of the disease caused by the mutation. On the contrary, the P2 mutation was classified as pathogenic in all the programs used.

**Table 2:** Features of new mutations found in patients 1 and 2.

Patient	Position				Prediction			
	Exon	Genomic Chr1 (GRCh38/hg38)	cDNA NM_000478.6	Protein P05186-1	MutPred	PROVEAN	Mutation Taster	CADD MSC:8.155
**P1**	2	g.21554098	c.17T>C	L6S	Probably benign Score: 0.478	Deleterious Score: -2,911	Polymorphism *p*-value: 0.99523	High Damage Prediction: 23.70
**P2**	6	g.21564063_21564065 CAC/-	c.498_500delCAC	T167del	Pathogenic Score: 0.7978	Deleterious Score: -14,023	Disease Causing *p*-value: 0.99999	High Damage Prediction: 19.90

### 3.3 Family segregation analysis

After identifying mutations in P1 and P2, serum ALP levels of close relatives were studied and those who presented persistently low levels underwent genetic study to investigate the origin of these mutations while those who presented serum ALP values within the normal range ​​were discarded from the study following the clinical algorithm criteria described by our group (4). **Table 3** shows the results for relatives of both patients affected by the new variants. In Family 1, I-2a, II-2a, and II-3a had the same mutation as P1, with consistently low levels of ALP activity (18, 20, and 25 IU/L, respectively). I-2a was treated with cholecalciferol and presented digestive disorders such as chronic diarrhea and diverticulitis. This patient also presented inflammatory diseases such as arteritis or polymyalgia rheumatica with current treatment. Regarding pathognomonic symptoms of HPP, this patient lost several teeth at an early age. II-2a presented with chronic gastritis, parathyroidism and a giant cell tumor affecting the little finger. Both I-2a and II-2a have elevated levels of interleukin 6 (IL6), indicating a pro-inflammatory state. II-3a had fibromyalgia, tooth infections, psoriatic arthritis, and other skeletal abnormalities detailed in **Table 3**. In Family 2, the P2 mutation came from II-1b, who also had low ALP activity (below 43 IU/L), tooth loss, and high blood pressure. It appears this variant was inherited from I-2b, who had a similar tooth loss condition associated with odontohypophosphatasia. All carriers of the new variants in the *ALPL* gene from both families presented vitamin D deficiency (<30 ng/mL). **Figure 2B** illustrates these findings in a family tree with clinical and sequencing results.

**Table 3:** Clinical results of relatives affected with HPP.

	I-2a	II-1a	III-1a	II-1b
*ALPL* mutation	c.17T>C (L6S)	c.17T>C (L6S)	c.17T>C (L6S)	c.498_500delCAC (T167del)
ALP Activity (33-98 IU/L females and 43-115 IU/L males)	18	20	32	32
Age (years)	79	51	50	51
BMI (Kg/m^2^)	27.5	22.86	34	31
Serum 25-vit D (30-50 ng/mL)	27.3	9.00	13.1	26.10
Protein c-reactive (0-5 mg/L)	4.1	1.00	6.7	1.90
Interleucin-6 (1.5-7 pg/mL)	12.1	12.30	–	1.50
Parathyroid Hormone (12-110 pg/mL)	43.2	321.80	–	42.80
Symptoms	Diverticulosis, constitutional syndrome, normochromic anemia, vitamin D deficiency, polymyalgia rheumatica, giant cell arteritis, chronic diarrhea, grade A esophagitis, carpal tunnel syndrome, type 2 diabetes, and tooth loss	Chronic Gastritis, vitamin D deficiency and giant cell tumor	Fibromyalgia, Psoriatic arthritis, tooth infection, neutrophilia, Carpal tunnel syndrome, Pelvic dysmetria, morbid obesity, vitamin D deficiency, spondyloarthritis with discarthrosis and HBP	Tooth loss, right knee pain, vitamin D deficiency and HBP

HBP, high blood pressure.

### 3.4 Multiple sequence alignment

To know the degree of conservation of the mutated amino acids, MSA was performed using TNSALP sequences. Twenty animal TNSALP sequences were chosen for MSA (**Supplementary Figure S1**).

The results obtained demonstrated that the beginning of the sequence is quite poorly conserved except for three amino acids, M^1^, L^6^ and L^8^, which match all the sequences analyzed. Regarding the mutation of P2, the T^167^ matches throughout all aligned sequences (**Figure 2C**).

### 3.5 3D modelling

**Figure 3:**
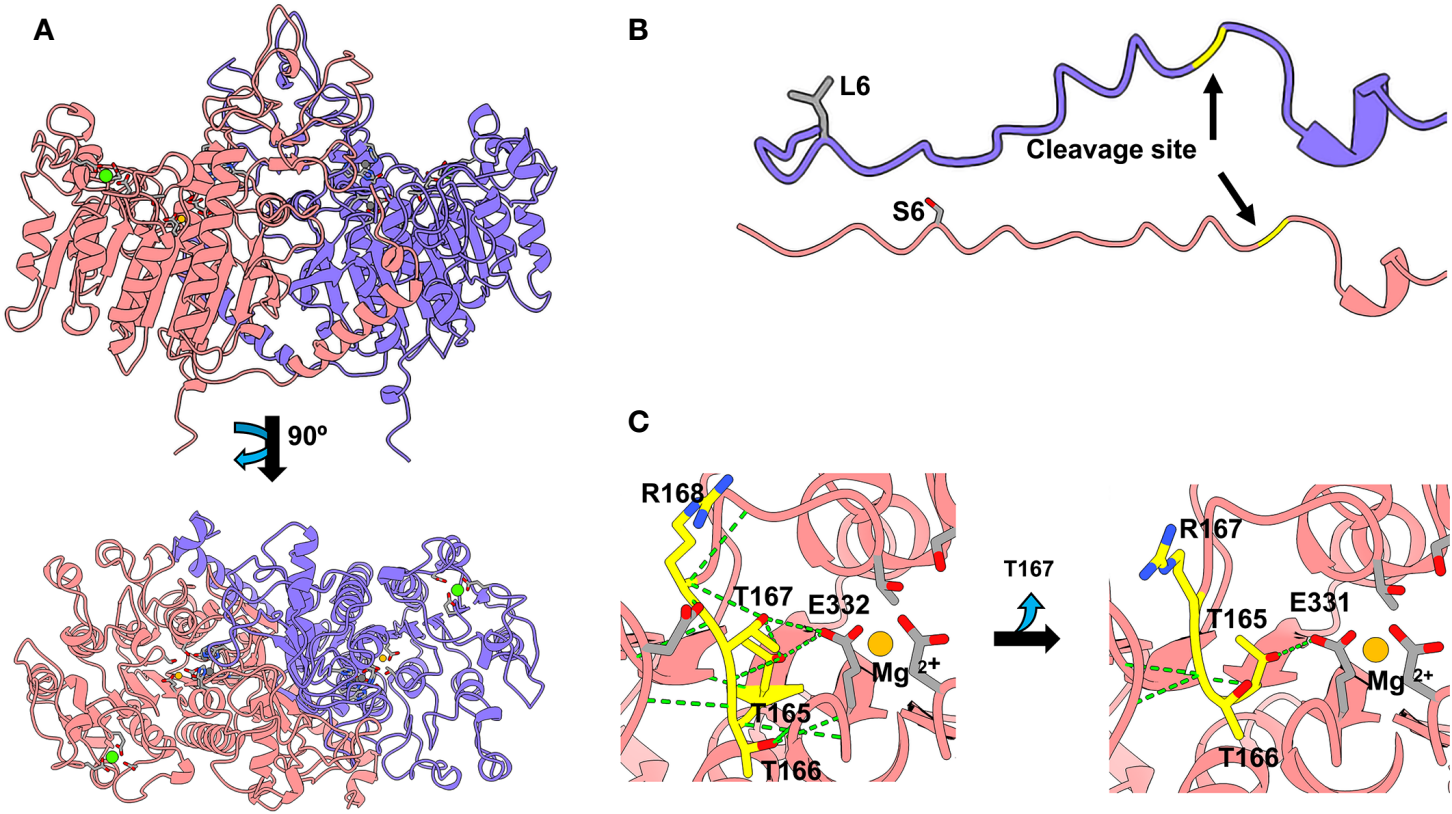
TNSALP 3D modelling based on the algorithm developed in AlphaFold2_advanced and visualized in Chimera X (Web References). **(A)** shows the visualization of TNSALP WT top and bottom structure. **(B)** shows the representation of the WT TNSALP and variant L6S signal peptide. **(C)** shows the representation of the structural characteristics of the T167del mutation.

After simulation in AlphaFold2_advanced, the highest-ranked predictions based on the pLDDT score were chosen for WT (**Figure 3A**) and both new variants.

L6S affected the signal peptide, while the catalytic core was not affected (**Figure 3B**). In contrast, T167del produces the shortening of a beta-loop in the vicinity of the active site of the protein. This loop is built by three consecutive threonines and one arginine (^165^TTTR^168^). T^165^ and T^167^ establish H-bonds with E^332^ which interacts directly with the Mg^2+^ of the active center. T^166^ generates hydrogen bonds with G^334^ and R^335^, forming an alpha helix above the catalytic site (**Figure 3C**). The shortening of the loop due to the deletion of T^167^ causes the loss of most of the hydrogen bonds, including those that affect the catalytic site.

### 3.6 Functional validation of mutations using a cell-based assay

**Figure 4:**
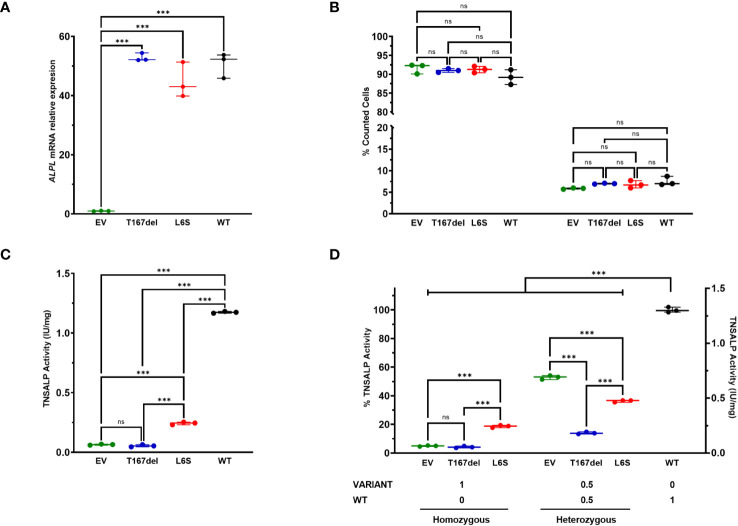
*In vitro* characterization of TNSALP activity of studied variants. Results are expressed as mean with standard deviation. ANOVA was used for comparisons between groups (***p<0.001). **(A)** shows relative mRNA expression after overexpression of new *ALPL* gene variants in HEK293T cells. The results were normalized using the housekeeping *RPL13*. **(B)** shows cell viability results obtained by flow cytometry. The results of survival and apoptosis were represented as a percentage concerning the total count of the cells in each culture. **(C)** shows TNSALP activity determination of the new *ALPL* variants. The quantitative results of the ALP assay were expressed in International Units per milligram of protein (IU/mg). **(D)** shows determination of the dominant negative effect of the study variants. Determinations were made by TNSALP activity and normalized per mg of protein. ns: not significant.

Results of *ALPL* gene expression by RT-qPCR showed that cells transfected with the mutated vectors and with the WT vector had a 45-55 fold change compared to EV (*p*<0.001). There was no significant difference between the mutations and WT (**Figure 4A**).

Cell survival was not compromised in any of the cases. **Figure 4B** shows that all populations had high survival rates (92-89%) and low apoptosis rates (5-7%). Furthermore, no statistically significant differences were observed between any of the study groups.

### 3.7 TNSALP activity

TNSALP activity in transfected cells showed a decrease in two variants compared to the WT protein. As shown in **Figure 4C**, the T167del variant had null activity, with no significant differences with respect to EV whereas the L6S variant had a statistically significant higher activity compared to EV and the T167del mutation.

Regarding the measurements of the TNSALP activity in the co-transfections, lower activity is observed in those in which the mutations were used compared to those that used EV (**Figure 4D**). In heterozygosity, EV: WT presents a percentage of activity of 53.06%, which represents half of the total activity obtained in cells transfected in homozygosis with the WT plasmid, while L6S: WT and T167del: WT in heterozygosis presented an average activity of 36.33. % and 13.97% respectively. These results confirm that although both variants generate a DNE, the T167del variant exerts a greater effect than the L6S variant on the WT monomer.

### 3.8 Cellular localization of TNSALP

The expression of TNSALP in the cell membrane was analyzed by flow cytometry in homozygosity and heterozygosity of each variant.

**Figure 5:**
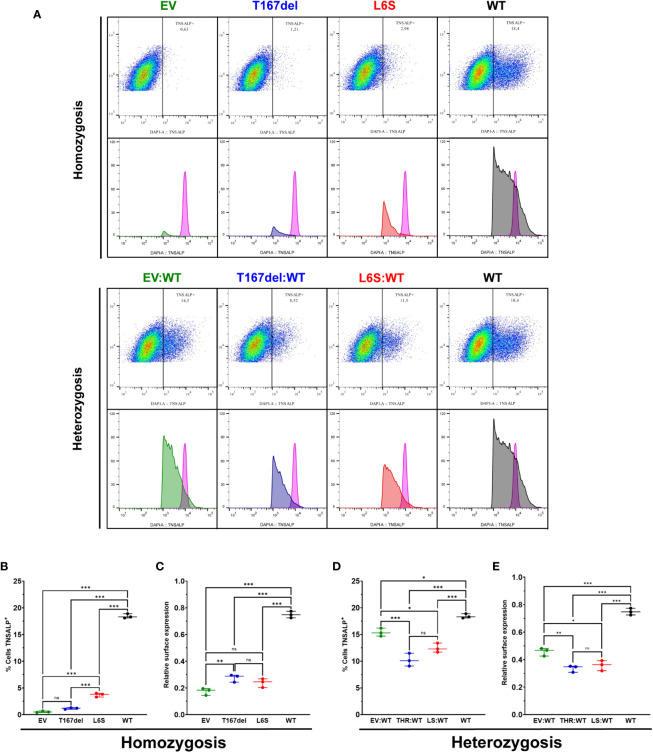
Determination of TNSALP localization on the cell surface of HEK293T cells. **(A)** shows an example of flow cytometry gating and histogram in cells homozygous and heterozygous for TNSALP variants. The internal control is represented by the histogram in pink. **(B, C)** show the percentage of TNSALP positive cells **(B)** and TNSALP relative expression on cell surface **(C)** in homozygosis. Results are expressed as mean with standard deviation. ANOVA was used for comparisons between groups (***p<0.001, **p<0.01, *p<0.05). **(D, E)** show the percentage of TNSALP positive cells **(D)** and TNSALP relative expression on cell surface **(E)** in heterozygosis. Results are expressed as mean with standard deviation. ANOVA was used for comparisons between groups (***p<0.001, **p<0.01, *p<0.05). ns: not significant.

**Figure 5A** shows the gating process for the selection of those cells that expressed TNSALP on the surface of the cell membrane for the homozygous and heterozygous. **Figure 5B** shows cells that expressed TNSALP on the surface under homozygous conditions. The cell population with the WT variant obtained a significantly higher percentage of cells that expressed TNSALP on the surface (18.43%), followed by the cell population that expressed the L6S variant (3.7%), while the cells that expressed the T167del variant (1.16%) did not present differences in the percentage of cells that expressed TNSALP compared to EV (0.53%). **Figure 5C** shows the amount of TNSALP on the cell surface of the different homozygous conditions. The WT variant expressed a significantly higher amount of surface protein (0.749) than the other variants. There were no differences between the populations that expressed the T167del (0.27) and L6S (0.24) variants; however, the cells that expressed T167del showed a greater amount of TNSALP on the surface than those cells that expressed EV (0.17).

Cells in heterozygosity had a significant increase in the percentage of cells expressing TNSALP on the cell surface. The EV: WT variant (15.4%) presented a percentage of cells that expressed TNSALP significantly higher than those cells heterozygous for the T167del: WT (10.23%) and L6S: WT (12.46%) variants; however, they presented a lower percentage of cells expressing TNSALP than cells homozygous for the WT variant (18.43%) (**Figure 5D**). Regarding antigenic density, the homozygous cells that expressed the WT variant (0.749) expressed a significantly higher amount of protein on the surface than those cells that expressed the variants in heterozygosis. Heterozygous cells that expressed EV: WT (0.46) showed significantly more surface protein than those cells that expressed the T167del:WT (0.336) and L6S:WT (0.358) variants (**Figure 5E**).

## 4 Discussion

In this study, two new, previously undescribed variants in the *ALPL* gene have been identified in two 15-year-old male patients, leading to the genetic screening of the patients’ relatives for better understanding and personalized management for affected families.

*In silico* predictions concluded that T167del is probably pathogenic while L6S does not present a clear consensus. The results of the alignment analysis indicate that the Met^1^, Leu^6^ and Leu^8^ amino acids for L6S variant and ^165^TTTR^168^ amino acids for T167del variant are highly conserved across all species included in this analysis (**Supplementary Figure S1**). These results besides 3D modeling (**Figures 3B, C**) suggest that these residues may play a major role in the protein function. We suggest that Met^1^, Leu^6^ and Leu^8^ amino acids in L6S variant could be involved in the cellular localization of the protein agreeing with the results obtained by Silvent et al. (35). For T167del variant T^167^ could play an important role as a stabilizer of the active site, since we found that it has a structural function providing stability to E^332^, a direct Mg^2+^ ligand that is part of the catalytic center.

Other mutations have been identified in the same positions as L6S variant (c.17T>A; L6*) (36) and T167del variant (c.500C>T; T167M) (37–39). For the first one, this variant is considered pathogenic; however, the effect of the mutation is not comparable to our identified variant since c.17T>A gives rise to a nonsense mutation resulting in a 6 amino acid peptide (36). For the second one, it has been associated with a severe pathogenic phenotype of HPP being described in patients with severe childhood HPP (37), patients with non-lethal perinatal HPP (38) and adult HPP (39). Based on our results and previous scientific literature, T167del variant has been classified as pathogenic.

The results of the *in vitro* TNSALP activity are consistent with the results observed at the clinical level. P1 presented significantly higher serum ALP activity (73 and 42 IU/L) than P2 (45 and 38 IU/L) in concordance with the *in vitro* results where a substantial decrease in TNSALP activity was observed in T167del compared to L6S (**Figure 4C**). The suspicion that both variants might be pathogenic is reinforced by the determination of their negative dominance over the WT monomer (**Figure 4D**). In both cases, TNSALP activity was lower than that obtained in the control group. The fact that the L6S allele presents a moderate dominance over the WT allele is consistent with the serum ALP activity of P1 being close to the reference range. Furthermore, it has been described that mutations with higher DNE are usually found in the crown domain, homodimeric interphase, or the area of the active center (26). This agrees with the results obtained for T167del (located close to the active center of the protein) which showed more severe DNE.

Our results showed retention in the efflux of protein to the membrane both in homozygosity and in heterozygosity due to the drastic reduction both in the amount of protein in the membrane and in the number of cells expressing TNSALP. In this context, the use of flow cytometry is a very useful tool to obtain relevant explanations about TNSALP cellular localization. In the case of the L6S variant, the same mutation has been described in gap junction protein beta 1, producing its accumulation at the intracellular level (40). We have observed a decrease not only in the percentage of cells that express TNSALP but also in the amount of protein in the membrane. Thus, the amino acid L^6^ could play an important role in the process of exporting TNSALP to the cell membrane which could explain the low antigenic density of TNSALP although the percentage of cells expressing this protein is higher. In the case of the T167del variant, the underlying mechanisms in the alteration of the cellular localization of the protein are unknown to date. However, due to the pathogenicity of the mutation, we suggest that the mutated protein could be taken to the proteasome for its complete degradation, which would explain the low activity and antigenic density of both homozygous and heterozygous.

Regarding clinical manifestations, in Family 1 there is a clear vitamin D deficiency in patients affected by L6S variant. Vitamin D deficiency is highly prevalent in HPP patients and should be treated with vitamin D supplements to avoid secondary hyperparathyroidism (41) as observed in II-2a. I-2a had tooth loss at an early age and a series of symptoms different from the pathognomonic symptoms of HPP such as digestive abnormalities (chronic diarrhea, esophagitis and diverticulitis) or inflammatory diseases (arteritis and polymyalgia rheumatica). II-2a presented chronic gastritis as a digestive symptom and giant cell tumor and hyperparathyroidism while II-3a suffered from neurological disorders such as fibromyalgia, immune disorders such as spondyloarthritis with dyscarthrosis, psoriatic arthritis and neutrophilia; skeletal disorders such as pelvic dysmetria and HBP (**Table 3**). P1 inherited some of these manifestations such as digestive disorders, vitamin D deficiency and HBP at an early age (**Table 1**). Regarding Family 2, both carriers of new T167del variant from Family 2 showed vitamin D deficiency. II-1b presented early tooth loss consistent with a phenotype of odontohypophosphatasia (**Table 3**) while P2 suffered from inverted psoriasis and Crohn’s disease inherited from II-2b (**Table 1**). The *in vitro* results of T167del variant besides to the immune disturbances, suggest that this HPP-related genotype could contribute to a worse prognosis of the comorbidities. In this context, some symptoms such as fibromyalgia (21, 39, 42, 43), digestive affections (44) or HBP (43) have been slightly linked to HPP; however, these complications as a symptom related to HPP have not been explored in depth to date. Some studies suggest that digestive alterations may be associated with disturbances in the immune system (45, 46). This hypothesis is strengthened by the clinical manifestations of P2 who has been diagnosed with Crohn’s disease and inverse psoriasis. Overall, 50% of the patients studied had some type of autoimmune/inflammatory disease in two unrelated families. This finding is reinforced by a recent study published by our research group, in which a prevalence of immune diseases in HPP patients of 37.5% was observed. This prevalence represents an incidence of autoimmune diseases 4-10 times higher than that described for the general population (3-8%). Autoimmune diseases found in the patients participants in this study mainly affect bones, skin and circulatory system (6).

Our hypothesis is that some of the patients´ symptoms in the context of inflammatory process, could be related to the role of TNSALP beyond bone metabolism. In this context, increased TNSALP activity during episodes of late-onset sepsis suggests its immunomodulatory role (47). In this line, TNSALP has ectonucleotidase capacity, hydrolyzing extracellular ATP or LPS that act on Toll-like receptor 4 which is responsible for the activation of the innate immune system by macrophages releasing proinflammatories cytokines as interleukins 6 and 8 (48). These molecules are also degraded by intestinal alkaline phosphatase (IAP), which has been described as a protein involved in the development of inflammatory bowel disease. In fact, both isoenzymes, seem to have some cross-talk, since mutations in gene encoding IAP (called ALPI) lead to upregulation of TNSALP expression in intestinal tissue (49). This suggests that there could be some type of compensation of alkaline phosphatase activity when one of these enzymes presents loss of function. Moreover, TNSALP also regulates purinergic signaling, whereby extracellular ATP and ADP trigger inflammation through nucleotide receptors. TNSALP breaks down ATP and ADP into AMP and adenine, halting inflammation and promoting anti-inflammatory responses through adenine receptors. Thus, TNSALP has been associated with protecting against inflammation in diseases and favoring intestinal microbial populations by its role in ATP/ADP hydrolysis (13). On the other hand, TNSALP has been reported to be expressed in phagocytes (50), neutrophils (51) and T lymphocytes. A study in mice showed that TNSALP is required for the complete stimulation of T lymphocytes and T-cell-dependent colitis (10). Given the known role of TNSALP in modulating inflammation and the immune response, a deficiency in TNSALP activity may have some involvement in dysregulation of the immune system and for hence, could contribute to the worsening of inflammatory diseases found in many cases, in HPP patients. In this line some clinical trials where TNSALP is being used as a treatment for acute kidney injury associated with sepsis have been tested (52).

The computational 3D modeling and alignment linked to clinical results and functional analyses, suggest that L6S variant could be classified as likely pathogenic associated with a mild HPP phenotype. Regarding T167del variant, all the data collected suggest the classification of this variant as likely pathogenic with a moderate phenotype.

These results highlight the importance of establishing HPP as a systemic pathology, not only related to bone mineralization disturbances. Currently, digestive and autoimmune disorders are considered as independent processes in HPP patients; however, our findings reveal that these processes could be related to ALP deficiency. However, the etiology of these comorbidities remains elusive. Additional investigations are necessary to comprehend the pathogenic mechanisms associated with HPP-related complications fully. In this context, it is essential to increase the cohort of patients to corroborate and reinforce the potential correlation between TNSALP levels and the worsening of digestive, autoinflammatory, and/or autoimmune manifestations. Another limitation of the study lies in the utilization of a predictive 3D model obtained through alpha fold. A recent publication has presented a crystallized structure of TNSALP (53), offering valuable insights for forthcoming research endeavors. On the other hand, although it is difficult to establish a gene-phenotypic relationship of each variant described in HPP due to the participation of several external factors that enhance phenotypic variability, it is important to identify and characterize new variants that serve as a starting point for future research and patient management.

## 5 Conclusions

In conclusion, we have identified two new previously undescribed variants that produce clinical manifestations of HPP more related to systemic diseases than to bone disorders. Considering the results shown in this study linked to previous scientific evidence, we suggest low ALP activity could be related to worsening of inflammatory/autoimmune disorders in HPP patients. In this study we show that both new identified mutations could be classified as likely pathogenic and have a DNE that affects both enzymatic activity and cell location through overexpression in HEK293T cells.

## Web references

Leiden Open Variation Database (LOVD), https://databases.lovd.nl/shared/genes/ALPL (May 12, 2022); The Genome Aggregation Database (gnomAD), https://gnomad.broadinstitute.org/gene/ENSG00000162551?dataset=gnomad_r3 (May 12, 2022); ALPL mutation Database https://alplmutationdatabase.jku.at/ (Nov 16, 2023); Kyoto Encyclopedia of Genes and Genomes (KEGG), https://www.genome.jp/entry/3.1.3.1 (June 24, 2022); ClinVar, https://www.ncbi.nlm.nih.gov/clinvar/ (May 12, 2022); PROVEAN, https://www.jcvi.org/research/provean (May 27, 2022); MutPred, http://mutpred.mutdb.org/(May 27, 2022); http://mutpred2.mutdb.org/mutpredindel/ (May 27, 2022); Mutation Taster https://www.mutationtaster.org/ (May 28,2022); Combined annotation-dependent depletion (CADD), https://cadd.gs.washington.edu/score (June 15, 2022); mutation significance cut-off (MSC), http://pec630.rockefeller.edu:8080/MSC/ (June 15, 2022); UniProt, https://www.uniprot.org/ (June 23, 2022); AlphaFold2_advanced, https://colab.research.google.com/github/sokrypton/ColabFold/blob/main/AlphaFold2.ipynb (June 23, 2022); VarSome, https://varsome.com/ (June 29, 2023); Human Protein Atlas, https://www.proteinatlas.org/ENSG00000162551-ALPL/tissue#expression_cluster (November 01, 2023).

## Data availability statement

The original contributions presented in the study are included in the article/**Supplementary Material**. Further inquiries can be directed to the corresponding authors.

## Ethics statement

The studies involving humans were approved by Research Ethics Committee of Granada Center (Project ID: 0777-M1-20 approved on 8 May 2019). The studies were conducted in accordance with the local legislation and institutional requirements. Written informed consent for participation in this study was provided by the participants’ legal guardians/next of kin.

## Author contributions

LM-H: Conceptualization, Data curation, Formal Analysis, Investigation, Methodology, Software, Writing – original draft, Writing – review & editing. MM-T: Conceptualization, Funding acquisition, Project administration, Supervision, Writing – review & editing, Resources. RS-D: Formal Analysis, Investigation, Methodology, Writing – review & editing. ÁJ-O: Formal Analysis, Investigation, Methodology, Software, Writing – review & editing. FA-V: Data curation, Formal Analysis, Investigation, Methodology, Software, Writing – review & editing. TG-C: Data curation, Investigation, Validation, Writing – review & editing. VC-B: Data curation, Investigation, Methodology, Validation, Writing – review & editing. SG-S: Investigation, Methodology, Validation, Writing – review & editing. JG-V: Conceptualization, Data curation, Validation, Writing – review & editing. CG-F: Conceptualization, Data curation, Funding acquisition, Investigation, Methodology, Supervision, Visualization, Writing – original draft, Writing – review & editing. BG-F: Conceptualization, Data curation, Funding acquisition, Investigation, Methodology, Project administration, Resources, Supervision, Visualization, Writing – original draft, Writing – review & editing.
