# Supplementary material for: Systemic effects of hypophosphatasia characterization of two novel variants in the ALPL gene
Source: Front Endocrinol (Lausanne). 2024 Jan 3;14:1320516. doi: 10.3389/fendo.2023.1320516 (PMC10792043; doi:10.3389/fendo.2023.1320516)
Supplement: Supplementary file 1 [file DataSheet_1.docx]

Table S1. Transcription variants containing the L6S mutation.

| NCBI Reference Sequence | Protein ID | Gene ID | CCDS | Name | Leu6 |
| --- | --- | --- | --- | --- | --- |
| NM_000478.6 | NP_000469.3 | 249 | CCDS217.1 | *Homo sapiens* alkaline phosphatase, biomineralization associated (ALPL), transcript variant 1, mRNA | YES |
| NM_001127501.4 | NP_001120973.2 | 249 | CCDS53275.1 | *Homo sapiens* alkaline phosphatase, biomineralization associated (ALPL), transcript variant 2, mRNA | NO |
| NM_001177520.3 | NP_001170991.1 | 249 | CCDS53274.1 | *Homo sapiens* alkaline phosphatase, biomineralization associated (ALPL), transcript variant 3, mRNA | NO |
| NM_001369803.2 | NP_001356732.1 | 249 | CCDS217.1 | *Homo sapiens* alkaline phosphatase, biomineralization associated (ALPL), transcript variant 4, mRNA | YES |
| NM_001369804.2 | NP_001356733.1 | 249 | CCDS217.1 | *Homo sapiens* alkaline phosphatase, biomineralization associated (ALPL), transcript variant 5, mRNA | YES |
| NM_001369805.2 | NP_001356734.1 | 249 | CCDS217.1 | *Homo sapiens* alkaline phosphatase, biomineralization associated (ALPL), transcript variant 6, mRNA | YES |
| XM_017000903.2 | XP_016856392.1 | 249 | - | PREDICTED: *Homo sapiens* alkaline phosphatase, biomineralization associated (ALPL), transcript variant X1, mRNA | NO |
| XM_054335748.1 | XP_054191723.1 | 249 | - | PREDICTED: *Homo sapiens* alkaline phosphatase, biomineralization associated (ALPL), transcript variant X1, mRNA | NO |

Table S2. Transcription variants containing the T167del mutation.

| NCBI Reference Sequence | Protein ID | Gene ID | CCDS | Name | Thr167 |
| --- | --- | --- | --- | --- | --- |
| NM_000478.6 | NP_000469.3 | 249 | CCDS217.1 | *Homo sapiens* alkaline phosphatase, biomineralization associated (ALPL), transcript variant 1, mRNA | YES |
| NM_001127501.4 | NP_001120973.2 | 249 | CCDS53275.1 | *Homo sapiens* alkaline phosphatase, biomineralization associated (ALPL), transcript variant 2, mRNA | YES |
| NM_001177520.3 | NP_001170991.1 | 249 | CCDS53274.1 | *Homo sapiens* alkaline phosphatase, biomineralization associated (ALPL), transcript variant 3, mRNA | YES |
| NM_001369803.2 | NP_001356732.1 | 249 | CCDS217.1 | *Homo sapiens* alkaline phosphatase, biomineralization associated (ALPL), transcript variant 4, mRNA | YES |
| NM_001369804.2 | NP_001356733.1 | 249 | CCDS217.1 | *Homo sapiens* alkaline phosphatase, biomineralization associated (ALPL), transcript variant 5, mRNA | YES |
| NM_001369805.2 | NP_001356734.1 | 249 | CCDS217.1 | *Homo sapiens* alkaline phosphatase, biomineralization associated (ALPL), transcript variant 6, mRNA | YES |
| XM_017000903.2 | XP_016856392.1 | 249 | - | PREDICTED: *Homo sapiens* alkaline phosphatase, biomineralization associated (ALPL), transcript variant X1, mRNA | YES |
| XM_054335748.1 | XP_054191723.1 | 249 | - | PREDICTED: *Homo sapiens* alkaline phosphatase, biomineralization associated (ALPL), transcript variant X1, mRNA | YES |

Table S3. Species name and sequence identification used for the MSA

| Specie | Name | Accesion |
| --- | --- | --- |
| *Homo Sapiens* | alkaline phosphatase, tissue-nonspecific isozyme isoform 1 preproprotein [*Homo sapiens*] | NP_000469.3 |
| *Pan troglodytes* | alkaline phosphatase, tissue-nonspecific isozyme isoform X1 [*Pan troglodytes*] | XP_016811306.1 |
| *Pan paniscus* | alkaline phosphatase, tissue-nonspecific isozyme isoform X1 [*Pan paniscus*] | XP_003813996.1 |
| *Gorilla gorilla* | alkaline phosphatase, tissue-nonspecific isozyme isoform X1 [*Gorilla gorilla gorilla*] | XP_055243051.1 |
| *Pongo abelii* | alkaline phosphatase, tissue-nonspecific isozyme isoform X1 [*Pongo abelii*] | XP_002811389.3 |
| *Macaca mulatta* | alkaline phosphatase, tissue-nonspecific isozyme precursor [*Macaca mulatta*] | NP_001253798.1 |
| *Mus musculus* | alkaline phosphatase [*Mus musculus*] | BAH03518.1 |
| *Rattus norvegicus* | alkaline phosphatase, tissue-nonspecific isozyme precursor [*Rattus norvegicus*] | NP_037191.2 |
| *Sus scrofa* | alkaline phosphatase, tissue-nonspecific isozyme isoform X2 [*Sus scrofa*] | XP_020953341.1 |
| *Canis lupus familiaris* | alkaline phosphatase, tissue-nonspecific isozyme isoform X1 [*Canis lupus familiaris*] | XP_038513640.1 |
| *Felis catus* | alkaline phosphatase, tissue-nonspecific isozyme isoform X1 [*Felis catus*] | XP_023113584.2 |
| *Bos taurus* | alkaline phosphatase, tissue-nonspecific isozyme precursor [*Bos taurus*] | NP_789828.2 |
| *Equus ferus caballus* | alkaline phosphatase, tissue-nonspecific isozyme isoform X1 [*Equus caballus*] | XP_005607435.1 |
| *Ovis aries* | alkaline phosphatase, tissue-nonspecific isozyme [*Ovis aries*] | XP_012008208.3 |
| *Gallus gallus* | alkaline phosphatase, tissue-nonspecific isozyme precursor [*Gallus gallus*] | NP_990691.2 |
| *Oxyura jamaicensis* | alkaline phosphatase, tissue-nonspecific isozyme [*Oxyura jamaicensis*] | XP_035168703.1 |
| *Xenopus laevis* | tissue-nonspecific alkaline phosphatase precursor [*Xenopus laevis*] | NP_001080895.1 |
| *Mauremys mutica* | alkaline phosphatase, tissue-nonspecific isozyme [*Mauremys mutica*] | XP_044851925.1 |
| *Anolis carolinensis* | PREDICTED: alkaline phosphatase, tissue-nonspecific isozyme [*Anolis carolinensis*] | XP_003229502.2 |
| *Alligator mississippiensis* | alkaline phosphatase, tissue-nonspecific isozyme [*Alligator mississippiensis*] | XP_006261757.2 |
| *Acipenser ruthenus* | alkaline phosphatase, tissue-nonspecific isozyme [*Acipenser ruthenus*] | XP_058890236.1 |

Table S4. List of primers used to study ALPL gene overexpression

| Name | Sequence |
| --- | --- |
| Forward ALPL | 5′-TGGCACCTGCCTTACTAACT-3′ |
| Reverse ALPL | 5′-CACGTTGGTGTTGAGCTTCT-3′. |
| Forward RPL13 | 5’-CGTAAGATCCGCAGACGTAAGGC-3’ |
| Reverse RPL13 | 5’-GGACTTGTTCCGCCTCCTCGGAT-3’ |

Figure S1. The whole alignment of TNSALP belonging to 150 different animals’ species

....|....| ....|....| ....|....| ....|....| ....|....| ....|....| ....|....| ....|....| ....|....| ....|....|

5 15 25 35 45 55 65 75 85 95

[Homo_sapiens] MISPFLVLAI GTCLTNSLVP EKEKDPKYWR DQAQETLKYA LELQKLNTNV AKNVIMFLGD GMGVSTVTAA RILKGQLHHN PGEETRLEMD KFPFVALSKT

[Pan_troglodytes] MISPFLVLAI GTCLTNSLVP EKEKDPKYWR DQAQETLKYA LKLQKLNTNV AKNVIMFLGD GMGVSTVTAA RILKGQLHHN PGEETRLEMD KFPFVALSKT

[Pan_paniscus] MISPFLVLAI GTCLTNSLVP EKEKDPKYWR DQAQETLKYA LKLQKLNTNV AKNVIMFLGD GMGVSTVTAA RILKGQLHHN PGEETRLEMD KFPFVALSKT

[Gorilla_gorilla_gorilla] MISPFLVLAI GTCLSNSLVP EKEKDPKYWR DQAQETLKYA LKLQRLNTNV AKNVIMFLGD GMGVSTVTAA RILKGQLHHN PGEETRLEMD KFPFVALSKT

[Pongo_abelii] MISPFLVLAI GTCLTNSLVP EKEKDPKYWR DQAQETLKYA LELQKLNTNV AKNVIMFLGD GMGVSTVTAA RILKGQLHHN PGEETRLEMD KFPFVALSKT

[Macaca_mulatta] MISPFLVLAI GTCLTNSLVP EKEKDPKYWR DQAQETLKYA LELQKLNTNV AKNVIMFLGD GMGVSTVTAT RILKGQLHHN PGEETRLEMD KFPFVALSKT

[Mus_musculus] MISPFLVLAI GTCLTNSFVP EKERDPSYWR QQAQETLKNA LKLQKLNTNV AKNVIMFLGD GMGVSTVTAA RILKGQLHHN TGEETRLEMD KFPFVALSKT

[Rattus_norvegicus] MILPFLVLAI GTCLTNSFVP EKEKDPSYWR QQAQETLKNA LKLQKLNTNV AKNIIMFLGD GMGVSTVTAA RILKGQLHHN TGEETRLEMD KFPFVALSKT

[Sus_scrofa] MISLFLVLAI GSCLTNSLVP EKEKDPKYWR DQAQQTLKNA LRLQTLNTNV AKNVIMFLGD GMGVSTVTAA RILKGQLHHK PGEETRLEMD KFPYVALSKT

[Canis_lupus] MISLFLVLVI GTCLTNSLVP EKEKDPKYWR DQAQQTLKYA LRLQNLNTNV AKNVIMFLGD GMGVSTVTAT RILKGQLHHN PGEETRLEMD KFPYVALSKT

[Felis_catus] MISPFLVLAI GTCLTNSLVP EKEKDPKYWR DQAQQTLKNA LRLQKLNTNV AKNVIMFLGD GMGVSTVTAA RILKGQLHHN PGEETRLEMD KFPYVALSKT

[Equus_caballus] MISPFLVLAI GTCLTNSLVP EKEKDPKYWR AQAQQTLQNA LRLQKLNTNV AKNIIMFLGD GMGVSTVTAA RILKGQLHNS PGEESRLEMD KFPFVALSKT

[Ovis_aries] MISPFLVLAI GTCLASSLVP EKEKDPKYWR DQAQQTLKNA LRLQTLNTNV AKNVIMFLGD GMGVSTVTAA RILKGQLHHN PGEETKLEMD KFPYVALSKT

[Gallus_gallus] MKAFLLTLLA QLCSA-SLVP EREKDPEYWR QQAQETLRDA LRLQHLNQNV AKNLILFLGD GMGVSTVTAA RILKGQLQHR KGEESLLEMD KFPYVALAKT

[Mauremys_mutica] MKALLLILLA EVCLA-SLVP EKEKNPQYWR NQAQQTLKTA LKLQQLNTNV AKNIILFLGD GMGVSTVTAA RILKGQLQNM KGEESLLEMD KFPYVALSKT

[Oxyura_jamaicensis] MKGFLLTLLV QLCSA-SLVP EKEKDPEYWR QQAQETLRDA LRLQRLNQNV AKNLILFLGD GMGVSTVTAA RILKGQLQNR KGEESLLEMD KFPYVALAKT

[Xenopus_laevis] MVKLWLLLLL GTLSESVTFP EQEKNPNYWR RQAQETLMNA LQLQNLNTNV AKNVIMFLGD GMGVPTVTAA RILKGQLAGQ PGEETQLEMD KFPHVALSKT

[Anolis_carolinensis] MTLLLLTLQL GLALA--FVP EKEKDPRYWR HQAQETLQEA LRLQELNTNV AKNVILFLGD GMGISTVTAS RILKGQLQNQ NGEESVLEMD RFPFVALSKT

[Alligator_mississippiensis] MTLLLLTLQL GLALA--FVP EKEKDPRYWR HQAQETLQEA LRLQELNTNV AKNVILFLGD GMGISTVTAS RILKGQLQNQ NGEESVLEMD RFPFVALSKT

[Phascolarctos_cinereus] MLLLLLGLMA GTCLS-SFVP EKEKNPQYWR DQAQRTLQHA LELQSLNTNI AKNVILFLGD GMGVSTVTAA RILKGQLHHM PGEDFQLEMD KFPFVALSKT

Clustal Consensus * * * .* *:*::* *** ***.** * *.** ** *: ***:*:**** ***:.****: ******* **: **** :**.***:**

....|....| ....|....| ....|....| ....|....| ....|....| ....|....| ....|....| ....|....| ....|....| ....|....|

105 115 125 135 145 155 165 175 185 195

[Homo_sapiens] YNTNAQVPDS AGTATAYLCG VKANEGTVGV SAATERSRCN TTQGNEVTSI LRWAKDAGKS VGIVTTTRVN HATPSAAYAH SADRDWYSDN EMPPEALSQG

[Pan_troglodytes] YNTNAQVPDS AGTATAYLCG VKANEGTVGV SAATERSRCN TTQGNEVTSI LHWAKDAGKS VGIVTTTRVN HATPSAAYAH SADRDWYSDN EMPPEALSQG

[Pan_paniscus] YNTNAQVPDS AGTATAYLCG VKANEGTVGV SAATERSRCN TTQGNEVTSI LHWAKDAGKS VGIVTTTRVN HATPSAAYAH SADRDWYSDN EMPPEALSQG

[Gorilla_gorilla_gorilla] YNTNAQVPDS AGTATAYLCG VKANEGTVGV SAATERSRCN TTQGNEVTSI LRWAKDAGKS VGIVTTTRVN HATPSAAYAH SADRDWYSDN EMPPEALSQG

[Pongo_abelii] YNTNAQVPDS AGTATAYLCG VKANEGTVGV SAATERSRCN TTQGNEVTSI LRWAKDAGKS VGIVTTTRVN HATPSAAYAH SADRDWYSDN EMPPEALSQG

[Macaca_mulatta] YNTNAQVPDS AGTATAYLCG VKANEGTVGV SAATERSRCN TTQGNEVTSI LRWAKDAGKS VGIVTTTRVN HATPSAAYAH SADRDWYSDN EMPPEALSQG

[Mus_musculus] YNTNAQVPDS AGTATAYLCG VKANEGTVGV SAATERTRCN TTQGNEVTSI LRWAKDAGKS VGIVTTTRVN HATPSAAYAH SADRDWYSDN EMPPEALSQG

[Rattus_norvegicus] YNTNAQVPDS AGTATAYLCG VKANEGTVGV SAATERTRCN TTQGNEVTSI LRWAKDAGKS VGIVTTTRVN HATPSAAYAH SADRDWYSDN EMPPEALSQG

[Sus_scrofa] YNTNAQVPDS AGTATAYLCG VKANEGTVGV SAATQRTQCN TTQGNEVTSI LRWAKDAGKS VGIVTTTRVN HATPSAAYAH SADRDWYSDN EMPPEALIQG

[Canis_lupus] YNTNAQVPDS AGTATAYLCG VKANEGTVGV SAATQRTQCN TTQGNEVTSI LRWAKDAGKS VGIVTTTRVN HATPSAAYAH SADRDWYSDN EMPPEALSQG

[Felis_catus] YNTNAQVPDS AGTATAYLCG VKANEGTVGV SAATQRTQCN TTQGNEVTSI LRWAKDSGKS VGIVTTTRVN HATPSAAYAH SADRDWYSDN EMPPEALSQG

[Equus_caballus] YNTNAQVPDS AGTATAYLCG VKANEGTVGV SAATQRTQCN TTQGNEVTSI LHWAKEAGKS VGIVTTTRVN HATPSAAYAH SADRDWYSDN EMPSEALSQG

[Ovis_aries] YNTNAQVPDS AGTATAYLCG VKANEGTVGV SAATQRSQCN TTQGNEVTSI LRWAKDAGKS VGIVTTTRVN HATPSASYAH SADRDWYSDN EMPPEALSQG

[Gallus_gallus] YNTNAQVPDS AGTATAYLCG VKANEGTVGV SAGVTRDRCN TTKGQEVTSI LRWAKDEGKA VGIVTTTRVT HATPSAAYAH SANRDWYSDG EMPLDALEGG

[Mauremys_mutica] YNTNAQVPDS AGTATAYLCG VKANEGTVGV SAAVTRAQCN TTAGNEVTSI LKWAKEAGKS VGIVTTTRVN HATPSAAYAH SANRDWYSDN EMPPEAIQQG

[Oxyura_jamaicensis] YNTNAQVPDS AGTATAYLCG VKANEGTVGV SAGVTRDRCN TTKGQEVTSI LRWAKDEGKA VGIVTTTRVT HATPSAAYAH SANRDWYSDG EMPPDALEGG

[Xenopus_laevis] YNTNAQVPDS AGTATAYLCG VKANEGTVGV NAAAVRNQCN TSKGNEVDSI MKWAKQAGKS VGVVTTTRIN HATPSAAYAH CVNRDWYSDQ EMPKEAVEQG

[Anolis_carolinensis] YNTNAQVPDS AGTATAYLCG VKANEGTVGV SAAVTRAQCN TTAGNHVTSI LRWAKDAGKS VGIVTTTRVN HATPSAAYAH SADREWYSDN EMPVEALQQG

[Alligator_mississippiensis] YNTNAQVPDS AGTATAYLCG VKANEGTVGV SAAVTRAQCN TTAGNHVTSI LRWAKDAGKS VGIVTTTRVN HATPSAAYAH SADREWYSDN EMPVEALQQG

[Phascolarctos_cinereus] YNTNAQVPDS AGTATAYLCG VKGNEGTLGV SAAVIRSQCN TTRGNEVTSI LRWAKDAGKS VGIVTTTRVN HATPSAAYAH SADRDWYSDN EMPTEALNQG

Clustal Consensus ********** ********** **.****:** .*.. * :** *: *:.* ** ::***: **: **:*****:. ******:*** ..:*:**** *** :*: *

....|....| ....|....| ....|....| ....|....| ....|....| ....|....| ....|....| ....|....| ....|....| ....|....|

205 215 225 235 245 255 265 275 285 295

[Homo_sapiens] CKDIAYQLMH NIRDIDVIMG GGRKYMYPKN KTDVEYESDE KARGTRLDGL DLVDTWKSFK PRYKHSHFIW NRTELLTLDP HNVDYLLGLF EPGDMQYELN

[Pan_troglodytes] CKDIAYQLMH NIRDIDVIMG GGRKYMYPKN KTDVEYESDE KARGTRLDGL DLVDTWKSFK PRHKHSHFIW NRTELLTLDP HNVDYLLGLF EPGDMQYELN

[Pan_paniscus] CKDIAYQLMH NIRDIDVIMG GGRKYMYPKN KTDVEYESDE KARGTRLDGL DLVDTWKSFK PRHKHSHFIW NRTELLTLDP HNVDYLLGLF EPGDMQYELN

[Gorilla_gorilla_gorilla] CKDIAYQLMH NIRDIDVIMG GGRKYMYPKN KTDVEYESDE KARGTRLDGL DLVDTWKSFK PRHKHSHFIW NRTELLTLDP HNVDYLLGLF EPGDMQYELN

[Pongo_abelii] CKDIAYQLMH NIRDIDVIMG GGRKYMYPKN KTDVEYEIDE KARGTRLDGL DLVDTWKSFK PRHKHSHFIW NRTELLTLDP HNVDYLLGLF EPGDMQYELN

[Macaca_mulatta] CKDIAYQLVH NIRDIDVIMG GGRKYMYPKN KTDVEYEIDE KARGTRLDGL DLVNIWKSFK PRHKHSHFIW NRTELLTLDP HNVDYLLGLF EPGDMEYELN

[Mus_musculus] CKDIAYQLMH NIKDIDVIMG GGRKYMYPKN RTDVEYELDE KARGTRLDGL DLISIWKSFK PRHKHSHYVW NRTELLALDP SRVDYLLGLF EPGDMQYELN

[Rattus_norvegicus] CKDIAYQLMH NIKDIDVIMG GGRKYMYPKN RTDVEYELDE KARGTRLDGL DLISIWKSFK PRHKHSHYVW NRTELLALDP SRVDYLLGLF EPGDMQYELN

[Sus_scrofa] CKDIAYQLMH NIRDIEVIMG GGRKYMFPKN RTDVEYEMDE KARGTRLDGL NLIDVWKSFK PRHKHSHYIW NRTELLALDP HTVDYLLGLF EPGDMQYELN

[Canis_lupus] CKDIAYQLMH NVKDIEVIMG GGRKYMFPKN RTDVEYEMDE KSRGTRLDGL NLIDIWKNFK PRHKHSHYVW NRTELLALDP YTVDYLLGLF EPGDMQYELN

[Felis_catus] CKDIAYQLMH NVRDIEVIMG GGRKYMFPKN RTDVEYEMDE KARGTRLDGL NLVDIWKSFK PRHKHSHYVW NRTELLTLDP YGVDYLLGLF EPGDMQYELN

[Equus_caballus] CKDIAYQLVH NIKDIEVIMG GGRKYMFPKN RTDVEYEMDE KARGTRLDGL NLIDIWKSFK PRHKHSHYIW NRTELLTLDP YTVDYLLGLF EPGDMQYELN

[Ovis_aries] CKDIAYQLMH NVKDIEVIMG GGRKYMFPKN RTDVEYELDE KARGTRLDGL NLVDIWKSFK PKHKHSHYVW NRTDLLALDP HTVDYLLGLF EPGDMQYELN

[Gallus_gallus] CKDIARQLVE NIPDIEVILG GGRKYMFPKN TSDVEYPQEE RHRGTRLDGK DLVQAWHDTK PAGKVAKYVW HRRELLALNV SRVDFLLGLF EPGDMVYELD

[Mauremys_mutica] CKDIAQQLFD NIPDIEVIMG GGRKYMFPKN TSDVEYQNDD KYSGTRLDGK NLIDVWRDKK PKDKNSRYVW NSIDLLSLNP MEVDFLLGLF EPIDMMYELD

[Oxyura_jamaicensis] CKDIARQLVE NIPDIEVILG GGRKYMFPKN ASDVEYPQEE KHRGTRLDRR DLVQAWHDTK PPGKVAKYVW HRRDLLALNL SRVDFLLGLF EPGDMVYELD

[Xenopus_laevis] CKDIAWQLMN NIPNIEVIMG GGRKYMFPKN TSDVEYPNDD KSSGTRLDGL NLTQIWLDQK PNKKAARYVW NREQLLSVNP QEVDHLLGLF EPVDMLYDLE

[Anolis_carolinensis] CKDIAHQLIE NVPDIEVIMG GGRKYMFPKN TSDVEYPEEE KYRGTRLDGR NLVEEWRRKK PSEKKAHYVW NQTELMKLDP SRVDYLLGLF EPIDLMYELN

[Alligator_mississippiensis] CKDIAHQLIE NVPDIEVIMG GGRKYMFPKN TSDVEYPEEE KYRGTRLDGR NLVEEWRRKK PSEKKAHYVW NQTELMKLDP SRVDYLLGLF EPIDLMYELN

[Phascolarctos_cinereus] CKDIAHQLIH NIPDIEVIMG GGRKYMFPKN TTDVEYGDDE KARGTRLDGQ FLVNVWKEKK PRNKNAHYVW NRMQLLELDL DRVDFLLGLF EPMDMTYELN

Clustal Consensus ***** **.. *: :*:**:* ******:*** :**** :: : ***** * . * * * * ::::* : :*: :: **.***** ** *: *:*:

....|....| ....|....| ....|....| ....|....| ....|....| ....|....| ....|....| ....|....| ....|....| ....|....|

305 315 325 335 345 355 365 375 385 395

[Homo_sapiens] RNNVTDPSLS EMVVVAIQIL RKNPKGFFLL VEGGRIDHGH HEGKAKQALH EAVEMDRAIG QAGSLTSSED TLTVVTADHS HVFTFGGYTP RGNSIFGLAP

[Pan_troglodytes] RNNVTDPSLS EMVVVAIQIL RKNPKGFFLL VEGGRIDHGH HEGKAKQALH EAVEMDRAIG QAGSLTSSED TLTVVTADHS HVFTFGGYTP RGNSIFGLAP

[Pan_paniscus] RNNVTDPSLS EMVVVAIQIL RKNPKGFFLL VEGGRIDHGH HEGKAKQALH EAVEMDRAIG QAGSLTSSED TLTVVTADHS HVFTFGGYTP RGNSIFGLAP

[Gorilla_gorilla_gorilla] RNNMTDPSLS EMVVVAIQIL RKNPKGFFLL VEGGRIDHGH HEGKAKQALH EAVEMDRAIG QAGSLTSSED TLTVVTADHS HVFTFGGYTP RGNSIFGLAP

[Pongo_abelii] RNNVTDPSLS EMVVVAIQIL RKNPKGFFLL VEGGRIDHGH HEGKAKQALH EAVEMDRAIG QAGSLTSSED TLTVVTADHS HVFTFGGYTP RGNSIFGLAP

[Macaca_mulatta] RNNVTDPSLS EMVVVAIQIL RKNPKGFFLL VEGGRIDHGH HEGKAKQALH EAVEMDRAIG QAGSMTSLED TLTVVTADHS HVFTFGGYTP RGNSIFGLAP

[Mus_musculus] RNNLTDPSLS EMVEVALQIL TKNPKGFFLL VEGGRIDHGH HEGKAKQALH EAVEMDQAIG KAGAMTSQKD TLTVVTADHS HVFTFGGYTP RGNSIFGLAP

[Rattus_norvegicus] RNNLTDPSLS EMVEVALRIL TKNPKGFFLL VEGGRIDHGH HEGKAKQALH EAVEMDEAIG KAGTMTSQKD TLTVVTADHS HVFTFGGYTP RGNSIFGLAP

[Sus_scrofa] RNNVTDPSLS EMVEMAIRIL IKNPKGFFLL VEGGRIDHGH HEGKAKQALH EAVEMDRAIE QAGSMTSVED TLTVVTADHS HVFTFGGYTP RGNSIFGLAP

[Canis_lupus] RNNVTDPSLS EMVEIAIKIL SKNPRGFFLL VEGGRIDHGH HEGKAKQALH EAVEMDRAIG KAGVMTSLED TLTVVTADHS HVFTFGGYTP RGNSIFGLAP

[Felis_catus] RNSTTDPSLS EMVEIAIKIL SKNPKGFFLL VEGGRIDHGH HEGKAKQALH EAVEMDQAIG RAGAMTSLED TLTIVTADHS HVFTFGGYTP RGNSIFGLAP

[Equus_caballus] RNNVTDPSLS EMVEMAIKIL SKNPEGFFLL VEGGRIDHGH HEGKAKQALH EVVEMDRAIG QAGDMTSQED TLTVVTADHS HVFTFGGYTP RGNSIFGLAP

[Ovis_aries] RNNVTDPSLS EMVEMAIRIL NKNPKGFFLL VEGGRIDHGH HEGKAKQALH EAVEMDQAIG QAGAMTSVED TLTVVTADHS HVFTFGGYTP RGNSIFGLAP

[Gallus_gallus] RNNETDPSLS EMVAVAIRML QKNPRGFFLL VEGGRIDHGH HEGKAKQALH EAVELDRAVG LAGRLTSPRD TLSVVTADHS HVFTFGGYTP RGNPIFGLAP

[Mauremys_mutica] RNVQTDPSLT EMVQVAIRIL QRNPRGFFLL VEGGRIDHGH HEGKAKQALH EAVEMDRAIG KAGDMTSLED TLTVVTADHS HVFTFGGYTP RGNPIFGLAP

[Oxyura_jamaicensis] RNNETDPSLT EMVAVAIRML QKNPRGFFLL VEGGRIDHGH HEGKAKQALH EAVELDRAIG LAARLTSPQD TLSVVTADHS HVFTFGGYTP RGNPIFGLAP

[Xenopus_laevis] RNTTMDPSLS EMVEAAIKIL GKNPMGFFLL VEGGRIDHGH HEGKANQALH EAVQMDIAIG VSGNMTSVED TLTVVTADHS HVFTFGGYTH RGNPIFGLAP

[Anolis_carolinensis] RNKETDPSLT EMVDVALKVL QKNPLGFFLL VEGGRIDHGH HEGKAKQALH EAVEMDKAIG LADRMTSTQD TLTVVTADHS HVFTFGGYTP RGNTIFGLAP

[Alligator_mississippiensis] RNKETDPSLT EMVDVALKVL QKNPLGFFLL VEGGRIDHGH HEGKAKQALH EAVEMDKAIG LADRMTSTQD TLTVVTADHS HVFTFGGYTP RGNTIFGLAP

[Phascolarctos_cinereus] RNNQTDPSLT EMVEVAIRIL RRNPKGFFLL VEGGRIDHGH HEGKAKQALH EAVEMDRAIG QAGTLTSLED TLTVVTADHS HVFTFGGYTP RGNSIFGLAP

Clustal Consensus ** ****: *** *:::* :** ***** ********** *****:**** *.*::* *: : :** .* **::****** ********* ***.******

....|....| ....|....| ....|....| ....|....| ....|....| ....|....| ....|....| ....|....| ....|....| ....|....|

405 415 425 435 445 455 465 475 485 495

[Homo_sapiens] MLSDTDKKPF TAILYGNGPG YKVVGGEREN VSMVDYAHNN YQAQSAVPLR HETHGGEDVA VFSKGPMAHL LHGVHEQNYV PHVMAYAACI GANLGHCAPA

[Pan_troglodytes] MLSDTDKKPF TAILYGNGPG YKVVGGEREN VSMVDYAHNN YQAQSAVPLR HETHGGEDVA VFSKGPMAHL LHGVHEQNYI PHVMAYAACI GANLDHCAPA

[Pan_paniscus] MLSDTDKKPF TAILYGNGPG YKVVGGEREN VSMVDYAHNN YQAQSAVPLR HETHGGEDVA VFSKGPMAHL LHGVHEQNYI PHVMAYAACI GANLDHCAPA

[Gorilla_gorilla_gorilla] MLSDTDKKPF TAILYGNGPG YKVVGGEREN VSMVDYAHNN YQAQSAVPLR HETHGGEDVA VFSKGPMAHL LHGVHEQNYI PHVMAYAACI GANLDHCAPA

[Pongo_abelii] MLSDTDKKPF TAILYGNGPG YKVVGGEREN VSMVDYAHNN YQAQSAVPLR HETHGGEDVA VFSKGPMAHL LHGVHEQNYI PHVMAYAACI GANLDHCASA

[Macaca_mulatta] MLSDTDKKPF TAILYGNGPG YKVVGGEREN VSMVDYAHNN YQAQSAVPLR HETHGGEDVA VFSKGPMAHL LHGVHEQNYI PHVMAYAACI GANLDHCAPA

[Mus_musculus] MVSDTDKKPF TAILYGNGPG YKVVDGEREN VSMVDYAHNN YQAQSAVPLR HETHGGEDVA VFAKGPMAHL LHGVHEQNYI PHVMAYASCI GANLDHCAWA

[Rattus_norvegicus] MVSDTDKKPF TAILYGNGPG YKVVDGEREN VSMVDYAHNN YQAQSAVPLR HETHGGEDVA VFAKGPMAHL LHGVHEQNYI PHVMAYASCI GANLDHCAWA

[Sus_scrofa] MVSDTDKKPF TAILYGNGPG YKVVGGEREN VSMVDYAHDN YQAQSAVPLR HETHGGEDVA IFARGPMAHL LHGVHEQNYI PHVMAYAACI GANRDHCASA

[Canis_lupus] MVSDTDKKPF TAILYGNGPG YKVVGGEREN VSMVDYAHNN YQAQSAVPLR HETHGGEDVA VFAKGPMAHL LHGVHEQNYI PHVMAYAACI GANQDHCASA

[Felis_catus] MVSDTDKKPF TSILYGNGPG YKVVGGEREN VSMVDYAHNN YQAQSAVPLR HETHGGEDVA VFAKGPMAHL LHGVHEQNYI PHVMAYAACI GANLDHCASA

[Equus_caballus] MVSDTDKKPF TAILYGNGPG YKVVGGEREN VSMVDYAHNN YQAQSAVPLR HETHGGEDVA VFAKGPMAHL LHGVHEQNYI PHVMAYAACI GANRDHCASA

[Ovis_aries] MVSDTDKKPF TAILYGNGPG YKVVGGEREN VSMVDYAHNN YQAQSAVPLR HETHGGEDVA VFAKGPMAHL LHGVHEQNYI PHVMAYAACI GANRDHCASA

[Gallus_gallus] MQSDVDRKPF TSILYGNGPG YKIVGGEREN VSAVDFAHAN YQAQAAVPLR QETHGGEDVA VFARGPMAHL LHGVHEQNYI PHAMAYAACI GPNRAHCSSA

[Mauremys_mutica] MHSDVDKMPF TSILYGNGPG YKIVAGEREN VSAVNYAHAN YQAQSAVPLR QETHGGEDVA IFAKGPMAHL LHGVHEQNYI PHVMAYAACI GQNTNHCSSG

[Oxyura_jamaicensis] MQSDVDRKPF TSILYGNGPG YKIVGGEREN VSAVDFAHAN YQAQSAVPLR QETHGGEDVA VFARGPMAHL LHGVHEQNYI PHAMAYAACI GPNRAHCNAA

[Xenopus_laevis] VPSDVDQKPF TSILYGNGPG YKLVNGQREN VSTVDFSHPN YLAQSAVPLR METHGGEDVA VFAKGPMAHL LHGVHEQNYI PHVMAYASCI GQNQDHCTSG

[Anolis_carolinensis] KESDVDKKPF TSILYGNGPG FRLVGGEREN VSSTNFTDAN YQAQSAVPLR METHGGEDVA VFAKGPMAHL LHGVHEQNYI PHVMAFAACI GENRDHCRSG

[Alligator_mississippiensis] KESDVDKKPF TSILYGNGPG FRLVGGEREN VSSTNFTDAN YQAQSAVPLR METHGGEDVA VFAKGPMAHL LHGVHEQNYI PHVMAFAACI GENRDHCRSG

[Phascolarctos_cinereus] MLSDTDKKPF TSILYGNGPG YKVVAGEREN VSTVDYAHDN YQAQSAVPLR HETHGGEDVA VFAKGPMAHL LHGVHEQNYV PHVMAYASCI GANKDHCQLS

Clustal Consensus **.*: ** *:******** :::* *:*** ** .:::. * * **:***** ********* :*::****** *********: **.**:*:** * * ** .

....|....| ....|....| ....|...

505 515 525

[Homo_sapiens] SSAGSL-AAG PLL--LALAL YPLSVLF-

[Pan_troglodytes] SSAGSL-AAG PLL--LALAL FPLSVLF-

[Pan_paniscus] SSAGSL-AAG PLL--LALAL FPLSVLF-

[Gorilla_gorilla_gorilla] SSAGSL-AAG PLL--LALAL FPLSVLF-

[Pongo_abelii] SSAGSL-AAG PLL--LALAL FPLSVLF-

[Macaca_mulatta] SSAGSL-AAG PLL--LPLAL FPLSILF-

[Mus_musculus] GSGSAP-SPG ALL--LPLAV LSLRTLF-

[Rattus_norvegicus] SSASSP-SPG ALL--LPLAL FPLRTLF-

[Sus_scrofa] SSSGSP-SPG PLL--LLLAL LPLGILF-

[Canis_lupus] SSAGGPSSPG PLL--LLLAL LPVGILF-

[Felis_catus] SSAGGP-SPG PLF--LLLAL PSLGILF-

[Equus_caballus] SLAGSP-SPG PLL--LLLAL LPLGILF-

[Ovis_aries] SSSSSP-SPG PLL--LLLAL LPLGGLF-

[Gallus_gallus] ARPAAT-AT- ------LLPV LLLLLLLC

[Mauremys_mutica] AQHSTV-GPL PFL-STLTSV VLLKFLF-

[Oxyura_jamaicensis] GRAAAA-SP- -----LLLPF LTLLLLLC

[Xenopus_laevis] KGNNGA-RTS LILVSVLLPL FSLQLFY-

[Anolis_carolinensis] APPRGPWGPL PAS-ASALAL LLARSLF-

[Alligator_mississippiensis] APPRGPWGPL PAS-ASALAL LLARSLF-

[Phascolarctos_cinereus] SAARL--GSQ PALFPGLLTV FLLLSLFF

Clustal Consensus . .. :

The symbols (*, : , . ) represent identical, conserved and semi-conserved substitutions respectively while the absence of a symbol represents a lack of amino acid characteristic conservation.
